# Conservation planning in agricultural landscapes: hotspots of conflict between agriculture and nature

**DOI:** 10.1111/ddi.12291

**Published:** 2014-12-26

**Authors:** Gorm E Shackelford, Peter R Steward, Richard N German, Steven M Sait, Tim G Benton

**Affiliations:** Faculty of Biological Sciences, University of LeedsLeeds, LS2 9JT, UK

**Keywords:** Buffer zones, conservation biogeography, countryside biogeography, ecological intensification, food security, land sparing, protected areas, sustainable intensification, systematic conservation planning, wildlife-friendly farming

## Abstract

**Aim:**

Conservation conflict takes place where food production imposes a cost on wildlife conservation and vice versa. Where does conservation impose the maximum cost on production, by opposing the intensification and expansion of farmland? Where does conservation confer the maximum benefit on wildlife, by buffering and connecting protected areas with a habitable and permeable matrix of crop and non-crop habitat? Our aim was to map the costs and benefits of conservation versus production and thus to propose a conceptual framework for systematic conservation planning in agricultural landscapes.

**Location:**

World-wide.

**Methods:**

To quantify these costs and benefits, we used a geographic information system to sample the cropland of the world and map the proportion of non-crop habitat surrounding the cropland, the number of threatened vertebrates with potential to live in or move through the matrix and the yield gap of the cropland. We defined the potential for different types of conservation conflict in terms of interactions between habitat and yield (potential for expansion, intensification, both or neither). We used spatial scan statistics to find ‘hotspots’ of conservation conflict.

**Results:**

All of the ‘hottest’ hotspots of conservation conflict were in sub-Saharan Africa, which could have impacts on sustainable intensification in this region.

**Main conclusions:**

Systematic conservation planning could and should be used to identify hotspots of conservation conflict in agricultural landscapes, at multiple scales. The debate between ‘land sharing’ (extensive agriculture that is wildlife friendly) and ‘land sparing’ (intensive agriculture that is less wildlife friendly but also less extensive) could be resolved if sharing and sparing were used as different types of tool for resolving different types of conservation conflict (buffering and connecting protected areas by maintaining matrix quality, in different types of matrix). Therefore, both sharing and sparing should be prioritized in hotspots of conflict, in the context of countryside biogeography.

## Introduction

From 2005 to 2050, demand for food could as much as double (Tilman *et al*., 2011). To meet this increase in demand, it has been suggested that there should also be an increase in supply, much of which would need to come from an increase in production (The Royal Society, 2009). However, this suggestion is controversial (Lang & Barling, 2012; Tomlinson, 2013). Such an increase in production, without an increase in distribution, accessibility and affordability, might meet the demands of the rich, but it would not meet the needs of the poor or the undernourished, and it would have a massive impact on the environment, without insuring food security or food sovereignty (Tilman *et al*., 2001; Tscharntke *et al*., 2012; Loos *et al*., 2014). Moreover, an increase in demand for food could be met, at least in part, by a decrease in demand for livestock feed and biofuel feedstock, and a decrease in waste, without the need for such a massive increase in production (Foley *et al*., 2011; Bajželj *et al*., 2014).

Agriculture has already done more damage to nature than any other human activity (Balmford *et al*., 2012), and therefore, many conservationists are opposed to an increase in production. However, in view of the ‘new productivism’ in agricultural policy (Horlings & Marsden, 2011; Fish *et al*., 2013), it looks to us as though an increase is likely to take place – possibly a doubling of agricultural production and possibly a redoubling of agribusiness-as-usual – if the new incentives for overproduction are not replaced with new and renewed incentives for conservation, sustainable production, waste reduction, equitable distribution, accessibility and affordability (Donald *et al*., 2002; Schmid *et al*., 2007; Henle *et al*., 2008; Fischer *et al*., 2012; Loos *et al*., 2014). Nevertheless, conservationists could reduce the environmental impacts of an increase in food production by answering two questions. Where would an increase in production do the most damage to conservation, and where would it do the least? In other words, where are there ‘hotspots’ of conflict between agriculture and nature, and where are there not? The resolution of these ‘conservation conflicts’ (Balmford *et al*., 2001; Henle *et al*., 2008; Dobrovolski *et al*., 2011; Redpath *et al*., 2013) could then be prioritized in the ‘hottest’ hotspots.

Fundamentally, these conflicts are driven by the expansion and intensification of agriculture (Lambin & Meyfroidt, 2011; Baudron & Giller, 2014; Laurance *et al*., 2014). Agricultural expansion takes place at the expense of biodiversity, as natural habitats are cleared to make space for farmland (Gibbs *et al*., 2010), and habitat loss will probably be the primary driver of biodiversity loss this century (Sala *et al*., 2000). Clearly, the ‘agricultural frontiers’ of the world are among the hottest hotspots of conflict between agriculture and nature, such as the Amazon and Congo basins, where farmland is being carved out of the wilderness (Phalan *et al*., 2013). However, agricultural expansion also takes place behind the front lines of these conservation conflicts, where farmland is being carved out of fragments of natural habitat, and where small and diversified farms are being enlarged and simplified, often accompanied by the unsustainable use of agrochemical inputs, irrigation water and soil, under the banner of ‘conventional’ agricultural intensification (Benton *et al*., 2003; Tscharntke *et al*., 2012).

Agricultural land has the potential to be a wildlife habitat, in and of itself, but it also has the potential to be a vital part of a wildlife-friendly ‘matrix’ of agricultural and natural habitat that buffers protected areas from edge effects and facilitates the movement of wildlife between protected areas (Pimentel *et al*., 1992; Ricketts, 2001; Hansen & DeFries, 2007; Perfecto & Vandermeer, 2010). In the emerging theory of ‘countryside biogeography’ (Daily, 1997), the habitability and permeability of the matrix are thought to be the main reasons that small protected areas on land – which were once thought of as ‘islands’ of habitat in an ‘ocean’ of uninhabitable farmland – have lower rates of local extinction, relative to large protected areas, than predicted by the theory of ‘island biogeography’ (Mendenhall *et al*., 2014). Therefore, the conservation of countryside biodiversity should not only be about restricting agricultural land use in strict protected areas, which has been the focus of ‘systematic conservation planning’ (Margules & Pressey, 2000), but it should also be about buffering and connecting these protected areas with a habitable and permeable matrix (Perfecto & Vandermeer, 2010). We suggest that the matrix should be the target of a new form of systematic conservation planning in agricultural landscapes – a method of identifying agricultural landscapes of especially high quality (not only as wildlife habitats, in and of themselves, but also as buffers and connectors of protected areas) and prioritizing the resolution of conservation conflicts in these landscapes.

Systematic conservation planning is most effective when the costs and benefits of land use are analysed and optimized (Naidoo *et al*., 2006). Around the world, many agricultural landscapes have wide ‘yield gaps’ (where actual crop yields are much lower than potential crop yields) (Foley *et al*., 2011; Mueller *et al*., 2012), and the closing of the widest yield gaps would confer the greatest benefits on global food production. However, the conservation costs of closing these yield gaps have only just begun to be assessed (Cunningham *et al*., 2013; Phalan *et al*., 2014). We suggest that these costs and benefits should be assessed not only in terms of food production, but also in terms of wildlife conservation and other ecosystem services that these agricultural landscapes could provide as ‘multiple-use modules’ (Noss & Harris, 1986), in which core protected areas could be buffered and connected by a wildlife-friendly matrix.

As a conceptual framework for this cost–benefit analysis, we suggest that the conservation value of a multiple-use module is a function of the quantity and quality of wildlife habitat in the matrix, the number of species that live in or move through the matrix and the conservation status of these species. We also suggest that the production value of a multiple-use module – and thus the potential for conservation conflict – is a function of the yield gap of the cropland (potential for intensification) and the quantity and quality of non-cropland in the agricultural matrix that could potentially be cleared to make space for new cropland (potential for expansion). As a proof of concept, we used this conceptual framework to search for hotspots of conflict between agriculture and nature, on the global scale. This enabled us to explore priorities for resolving different types of conservation conflict in different places, and it could possibly enable us to steer an increase in food production towards places with low potential for conservation conflict (but only if an increase must take place).

## Methods

We used a map of global land cover to randomly sample the agricultural landscapes of the world (see Figs S1 & S2 in Supporting Information for graphical abstracts of these methods). Sampling points were restricted to land that was classified as cropland. For each point, (1) we used the GlobCover 2009 map (raster data with a resolution of about 300 m at the equator) (ESA & UCL, 2010) to calculate the proportion of non-crop habitat within 2 km of that point (see Appendix S1 for the classification of habitat in GlobCover), (2) we used the IUCN Red List of Threatened Species™ maps (vector data) (BirdLife International & NatureServe, 2012; IUCN, 2012) to calculate the number of ‘threatened’ and ‘Near-Threatened’ species of vertebrates (amphibians, birds, mammals and reptiles) with ranges that included that point (species with potential to live in or move through the matrix) and (3) we used the Global Agro-Ecological Zones (GAEZ) maps (raster data with a resolution of about 10 km at the equator) (IIASA/FAO, 2012) to measure the ratio of actual to potential yield (the yield gap). We deleted points that had no data on yield and points that were within protected areas with restrictions on agriculture, as defined by the GAEZ classification of data from the World Database on Protected Areas.

We then used these data points on non-crop habitat (*h*), vertebrate species (*s*) and relative yield (*y*) to map the potential for conservation conflict (*c*) on the global scale. We defined *c* as a function of *h*, *s* and *y* (Table 1), and we assumed that interactions between habitat and yield would result in different types of conflict. For example, we assumed that landscapes with high amounts of habitat and low yields, where an increase in food production could come from both expansion and intensification, would have a different type of conflict (Type III conflict in Table 1) than would landscapes with low amounts of habitat and low yields, where an increase could come only from intensification (Type II conflict). We then made heatmaps of the potential for these different types of conflict. Because of the latitudinal gradient in species richness (Whittaker *et al*., 2001), which is a source of bias towards high *c* at low latitudes, we also calculated *c* as a function of habitat and yield only (not species). We made heatmaps by interpolating *c* onto a 5-arc-min grid (a resolution of about 10 km at the equator, for comparison with the GAEZ maps) and then deleting pixels that did not have data on relative yields (GAEZ), pixels that were in protected areas with restrictions on agriculture (GAEZ) and pixels that were < 1% cropland (calculated from GlobCover).

**Table 1:** Potential for conservation conflict (*c*), as a function of habitat (*h*), species (*s*) and yield (*y*). For example, we suggest that the potential for Type III conflict is highest in landscapes with the highest amounts of habitat, highest numbers of species and lowest yields. Thus, *c* is maximized as *h × s ×* (1 − *y*) is maximized. These variables (*h*, *s* and *y*) could be given equal or unequal weights, based on the circumstances of the conflict, and thus, we use the tilde (∼) to suggest that these functions are approximations of the potential for conflict, not equations. For each variable (habitat, species and yield), the measured value at each data point (Data S1) was divided by the maximum value at all data points, and it was thereby transformed into a proportional variable (*h*, *s* and *y*). Therefore, 1 *− h* and 1 *− y* approach 0 as *h* and *y* approach 1

Type	Habitat (*h*)	Species (*s*)	Yield (*y*)	Potential for conflict (*c*)	Source of conflict
I	High	High	High	Max (*c*) ∼ max (*h* × *s* × *y*)	Expansion
II	Low	High	Low	Max (*c*) ∼ max ((1 − *h*) × *s* × (1 *− y*))	Intensification
III	High	High	Low	Max (*c*) ∼ max (*h* × *s* × (1 − *y*))	Both expansion and intensification
IV	Low	High	High	Max (*c*) ∼ max ((1 − *h*) × *s* × *y*)	Neither expansion nor intensification

We took a closer look at Type III conflict, which we regarded as the highest priority for conflict resolution (both expansion and intensification as a source of conflict). We classified each point as either a ‘case’ or a ‘control’ (Table 2), based on its potential for Type III conflict. For example, in analysis H1, points with *c*-values > 98% of all *c-*values were defined as cases, and other points were defined as controls. We then used spatial scan statistics to search for ‘hotspots’ of Type III conflict. Spatial scan statistics are usually used to search for significant spatial clusters of disease or crime (hence the terms ‘case’ and ‘control’), but we used them to search for significant spatial clusters of agricultural land with potential for conservation conflict. We used SaTScan™ (Kulldorff, 2013). For each data point, we searched for nearby data points (the ‘search area’ was a circle with a radius of 100, 200 or 400 km around the data point) and we calculated the proportion of data points that were cases in each search area. We defined ‘hotspots’ as search areas in which the proportion of cases was significantly higher than expected (*P* < 0.05), based on the proportion of cases in all search areas (Bernoulli models in SaTScan™).

**Table 2:** A data point was defined as either a case or a control, based on its high potential for conservation conflict (*c*) or its low potential for conservation conflict (*i*). For example, for hotspot analysis H3, only data points < 25 km from protected areas were analysed: a data point was either defined as a case if its *c-*value was > 98% of all *c-*values in that analysis, or else it was defined as a control; its *c-*value was calculated from *h*, *s* and *y* (as opposed to *h* and *y* only), using the formula for Type III hotspots; and its *h-*value was calculated using all non-crop habitat (as opposed to either grassland or woodland). For Type III hotspots, *c* = *h × s ×* (1 − *y*), and for Type I coldspots, *i* = (1 − *h*) *×* (1 − *s*) *×* (1 − *y*)

Hotspots	*h*	*c* (%)	Type	Protected areas	Coldspots	*h*	*i* (%)	Type	Protected areas
H1	Non-crop	> 98	III (*h*, *s*, *y*)	Any distance	C1	Non-crop	> 98	I (*h*, *s*, *y*)	Any distance
H2	Non-crop	> 95	III (*h*, *s*, *y*)	Any distance	C2	Non-crop	> 95	I (*h*, *s*, *y*)	Any distance
H3	Non-crop	> 98	III (*h*, *s*, *y*)	Points < 25 km	C3	Non-crop	> 98	I (*h*, *s*, *y*)	Points > 25 km
H4	Grassland	> 98	III (*h*, *s*, *y*)	Any distance	C4	Grassland	> 98	I (*h*, *s*, *y*)	Any distance
H5	Woodland	> 98	III (*h*, *s*, *y*)	Any distance	C5	Woodland	> 98	I (*h*, *s*, *y*)	Any distance
H6	Non-crop	> 98	III (*h*, *y*)	Any distance	C6	Non-crop	> 98	I (*h*, *y*)	Any distance

We also took a closer look at Type I conflict (expansion, but not intensification, as a source of conflict). We suggest that the potential for Type I conflict is lowest in landscapes with the lowest amounts of habitat (no potential for expansion), the lowest numbers of species and the lowest yields (potential for intensification). If an increase in food production is inevitable, then ‘coldspots’ of Type I conflict could be the landscapes that are most beneficial for intensification (potential to close the widest yield gaps) and least costly for conservation (potential to threaten the fewest species and the lowest amounts of habitat, if the local intensification of cropland causes the local expansion of cropland into non-crop habitat, by means of the mechanism known as the ‘rebound effect’ or the ‘Jevons paradox’) (Ewers *et al*., 2009; Lambin & Meyfroidt, 2011; Phelps *et al*., 2013). Therefore, without advocating an increase in food production, we used spatial scan statistics to search for coldspots of Type I conflict, as potential hotspots for sustainable intensification. Instead of searching for low *c-*values (Table 1), we searched for high *i-*values (‘*i*’ for ‘intensification’), where max (*i*) ∼ max ((1 − *h*) × (1 − *s*) × (1 − *y*)), because *i* is maximized only if *h*, *s* and *y* all have low values, whereas *c* is minimized if any one of *h*, *s* or *y* is equal to zero, even if the other two have high values.

To test the sensitivity of these assumptions (H1 and C1 in Table 2), we also searched for hotspots and coldspots under other sets of assumptions (H2–H6 and C2–C6 in Table 2). For example, in one set of sensitivity analyses (H4 and C4), we used the proportion of grassland within 2 km to calculate *h*, instead of the proportion of all non-crop habitat (which we defined as grassland + woodland), because fragments of grassland in the agricultural matrix could have different values as buffers and connectors of woodland protected areas than would fragments of woodland, and vice versa (Ricketts, 2001). In all sets of analyses, we used search areas of different radii (100, 200 or 400 km), to test for sensitivity to conservation planning on different scales. We then looked for areas where hotspots or coldspots were found in all analyses (the ‘hottest’ hotspots or ‘coldest’ coldspots).

## Results

**Figure 1:**
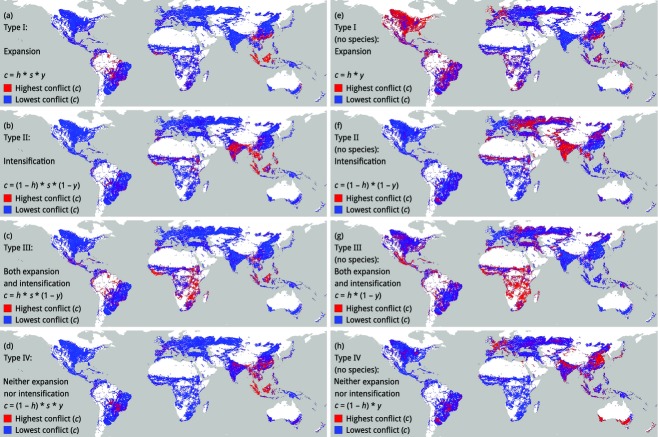
Heatmaps of the potential for conservation conflict (*c*), as a function of the proportion of non-crop habitat (*h*) within 2 km of cropland, the number of ‘threatened’ and ‘Near-Threatened’ species (*s*) of amphibians, birds, mammals and reptiles with potential to live in or move through cropland, and the relative yield (*y*) of cropland (panels a–d), or, as above, but as a function of habitat (*h*) and yield (*y*) only, not species (*s*) (panels e–h).

Sampling the cropland of the world resulted in 60405 data points (Data S1). Globally, cropland was surrounded by 44 ± 28% non-crop habitat within 2 km [mean ± standard deviation (SD)], it was potentially lived in or moved through by 11 ± 9 ‘threatened’ and ‘Near-Threatened’ vertebrate species (mean ± SD) and its actual yield was about 35% of its potential yield (Table 3). On heatmaps of the potential for conservation conflict (Fig.1a–d), the different types of conflict had distinct global distributions. For example, India was a hotspot of Type II and Type IV conflict, but not Type I or Type III conflict, whereas Indonesia and Malaysia were hotspots of all types of conflict. Therefore, on the global scale, there seemed to be potential to differentiate between regions with different types of conflict. However, the latitudinal gradient in species richness affected the global distribution of hotspots, some of which shifted to higher latitudes when *c* was calculated only from habitat and yield (not species) (Fig.1e–h). For example, in Fig.1(f–g), Indonesia and Malaysia were not hotspots of Type II or Type III conflict, and large parts of Eurasia and North America, which were coldspots in Fig.1(a–d), were hotspots in Fig.1(e–h).

**Table 3:** Comparison of data points in the hottest hotspots (H1–H5), the coldest coldspots (H1–H5) and the world: the number of cropland points (*N*), the percentage of non-crop habitat within 2 km of the average point (Habitat), the number of ‘threatened’ and ‘Near-Threatened’ vertebrate species with ranges that included the average point (Species) and the relative yield of the average point, both as a percentage of its potential yield (Yield) and also as its Global Agro-Ecological Zones (GAEZ) yield category (GAEZ), in which 1 is the lowest yield and 7 is the highest yield [average values are shown as mean ± standard deviation (SD)]. Comparisons between ‘spot’ averages and global averages were made using *t-*tests in which *t* = (spot mean – world mean)/(spot SD/√ spot *N*) and degrees of freedom = spot *N* − 1. Because of the high sample sizes (*N*), the *P*-values for all comparisons between spot averages and global averages were significant (*P* < 0.0001), and therefore, no *P-*values are shown in the table

Points	Search	*N*	Habitat (%)	Species	Yield (%)	GAEZ
H1–H5	100 km	490	72 ± 10	26 ± 9	15	2.3 ± 0.5
	200 km	1101	71 ± 13	23 ± 8	15	2.3 ± 0.5
	400 km	2539	70 ± 15	19 ± 7	16	2.4 ± 0.6
C1–C5	100 km	2495	27 ± 24	10 ± 3	13	2.2 ± 0.7
	200 km	5071	31 ± 25	10 ± 4	16	2.4 ± 0.8
	400 km	9855	34 ± 26	11 ± 5	20	2.7 ± 0.8
World	NA	60405	44 ± 28	11 ± 9	35	3.7 ± 1.2

**Figure 2:**
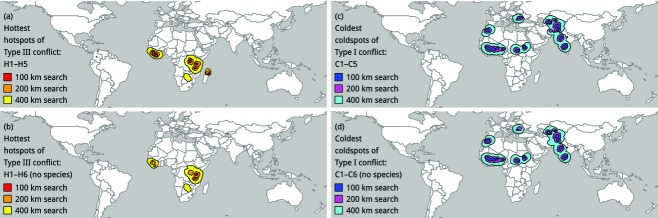
The hottest hotspots of Type III conflict and the coldest coldspots of Type I conflict. The hottest hotspots are the intersections between all of the hotspots (Fig. S3) that resulted from (a) analyses H1–H5 or (b) analyses H1–H6, which included the analysis (H6) that was not based on species. The coldest coldspots are the intersections between all of the coldspots (Fig. S3) that resulted from (c) analyses C1–C5 or (d) analyses C1–C6, which included the analysis (C6) that was not based on species.

These heatmaps offer some insight into the distributions of different types of conservation conflict, but the visual interpretation of these heatmaps is sensitive to the density of cropland, and it is subjective. By comparison, the statistical interpretation of the underlying data points, by means of spatial scan statistics, is not sensitive to the density of cropland, and it is not as subjective. In the strict consensus of analyses H1–H5, the hottest hotspots (Fig.2a) were all in sub-Saharan Africa, in three subregions: (1) West Africa, (2) Eastern and Southern Africa and (3) Madagascar. In the strict consensus of analyses C1–C5, the coldest coldspots (Fig.2c) were widespread, in five regions: (1) the Sahel region of sub-Saharan Africa, (2) North Africa, (3) Eastern Europe, (4) Central Europe and (5) South Asia. In the strict consensus of analyses H1–H6 or C1–C6, which included the analyses that used only habitat and yield (not species) to calculate the potential for conflict (H6 or C6), the results were surprisingly similar to those from the analyses that used habitat, species and yield (Fig.2), but we note that there were no hotspots in Madagascar and fewer hotspots throughout sub-Saharan Africa. Thus, the effects of the latitudinal gradient in species richness were accounted for in the hottest hotspots and coldest coldspots (see Fig. S3 for hotspots and coldspots from each analysis, H1–H6 and C1–C6).

In the hottest hotspots, cropland was surrounded by 72 ± 10% non-crop habitat within 2 km (mean ± SD), it was potentially lived in or moved through by 26 ± 9 ‘threatened’ and ‘Near-Threatened’ vertebrate species (mean ± SD) and its actual yield was about 15% of its potential yield (100 km search areas; Table 3). All of these measurements were significantly different from the global average, and this was also the case for all of the hottest hotspots and coldest coldspots (100–400 km search areas; Table 3). In the hottest hotspots, cropland had about 55–58% lower yield, was surrounded by 59–63% more habitat and was potentially lived in or moved through by 67–135% more species than the global average. In the coldest coldspots, cropland had about 44–63% lower yield, was surrounded by 24–38% less habitat and was potentially lived in or moved through by 5–14% fewer species than the global average.

## Discussion

Recent debate about the resolution of conservation conflict has been framed in terms of ‘land sharing’ (extensive agriculture that is wildlife friendly) versus ‘land sparing’ (intensive agriculture that is less wildlife friendly but also less extensive) as methods of growing the most food while doing the least damage to nature (Green *et al*., 2005; Phalan *et al*., 2011). It has been concluded that both sharing and sparing could be useful tools for conflict resolution (Hodgson *et al*., 2010; Tscharntke *et al*., 2012; Baudron & Giller, 2014; Fischer *et al*., 2014) However, we suggest that what is needed now is an evidence-based framework for deciding where to implement these tools, with limited amounts of time, money and land, and deciding how to use these tools to build resilience into the conservation planning system, by buffering and connecting protected areas with habitable and permeable agricultural landscapes.

If the debate between land sharing and land sparing were framed in these terms – that is, in terms of countryside biogeography – then the question would not be *whether* to share land or spare land, but *where* to share and where to spare, to maintain the habitability and permeability of the agricultural matrix. The answer to this question would depend upon the type of conservation conflict (expansion, intensification, both or neither). For example, in hotspots of Type IV conflict (low habitat, high species, high yield), neither would there be much land to spare, nor would there be a lot potential for increased yield to spare land elsewhere, and therefore land sharing could be a higher priority in these hotspots. However, our aim here is not to suggest that sharing should be a higher priority than sparing, or vice versa, as a resolution to any particular type of conservation conflict. Instead, our aim is to suggest that both sharing and sparing should be higher priorities in hotspots of conservation conflict than they should be in agricultural landscapes with lower potential for conflict. Therefore, our aim is to suggest that hotspots of conservation conflict could and should be defined and identified.

The present search for hotspots is only a proof of concept, and future research is needed to further develop this concept and to search for hotspots on scales that are appropriate for conflict resolution. Conservation planning on the global scale has the potential to confer greater benefits and impose lesser costs on nature than conservation planning on finer scales, if the costs and benefits of agriculture are also addressed (Dobrovolski *et al*., 2014), and therefore, the global scale could be an ideal starting point for conservation planning in agricultural landscapes. Conservation and production plans on the national scale have led to the ‘exportation’ of conservation conflicts to developing nations, through the importation of agricultural products by developed nations (Lambin & Meyfroidt, 2011), and thus, the plans that are made on the national scale are not independent of trade on the global scale. However, agricultural, ecological, economic, political and social processes take place on multiple scales and have multiple stakeholders (Sayer *et al*., 2013), and therefore, we suggest that hotspots of conservation conflict should be defined and identified on multiple scales, from local to global (Moilanen & Arponen, 2011; Gonthier *et al*., 2014), in the context of global trade and the need for local food security and food sovereignty (‘distributive’ and ‘procedural’ justice) (Loos *et al*., 2014). In this complex context, our definition of conservation conflict, in terms of habitat, species and yield only, is obviously an oversimplification. Nevertheless, the limitations of the present search should be seen as possibilities for future research in multiple fields, under multiple sets of assumptions about the value of conservation and production.

For example, we assumed that agricultural landscapes with the most habitat had the highest conservation value (hotspots of Type III conflict). In future research, it could be assumed that landscapes with the least habitat have the highest conservation value, because they could be the last refuges of endemic species, and indeed, ‘biodiversity hotspots’ have been identified as landscapes that have lost at least 70% of their natural habitat (Myers *et al*., 2000). However, we assumed that agricultural landscapes should not be replacements for protected areas, and therefore, they should not be evaluated in terms of unprotected species that they could protect on their own, but in terms of species that are nominally protected now (in protected areas) but would not be effectively protected in the future, if these protected areas were to become isolated in an ‘ocean’ of uninhabitable and impermeable agriculture. The effectiveness of protected areas depends upon the area of unprotected habitat in the landscapes that surround them (Wiersma *et al*., 2004), and thus, we assumed that agricultural landscapes with the most habitat had the highest conservation value. Therefore, hotspots of Type III conflict are ‘proactive’ as opposed to ‘reactive’ (Dobrovolski *et al*., 2011; Phalan *et al*., 2013). However, in future research, it could be assumed that ‘reactive’ conflicts over low levels of habitat (such as Type II and Type IV conflict) should be higher priorities.

We also assumed that agricultural landscapes with the most species had the highest conservation value. This is ethically utilitarian (‘the greatest happiness of the greatest number’), and it was based on threat and vulnerability, but other methods of assessment could be used, such as those based on complementarity, representativeness or any of the core methods of systematic conservation planning (Kukkala & Moilanen, 2013). As opposed to endemism, it could also be assumed that ‘cosmopolitanism’ should be a high priority for conservation planning in agricultural landscapes, because species with wide ranges could have high vulnerability to low matrix quality. However, the extinction of the passenger pigeon, which was widely ranging, but ‘endemic’ to only one type of widely ranging habitat (Bucher, 1992), exemplifies the limitations of such assumptions.

Considering the costs that some species impose on agriculture (such as elephants that raid crops or lions that kill livestock) and the benefits that some species confer on agriculture (such as bees that pollinate crops and wasps that kill crop pests), it could be assumed that potential for conservation conflict is highest where the perceived costs outweigh the perceived benefits by the most, and where the species that impose these costs are species of the greatest conservation concern. Research on pollination and pest control has shown that both of these ecosystem services are enhanced by high proportions of non-crop habitat (Chaplin-Kramer *et al*., 2011; Garibaldi *et al*., 2011), and indeed, the standard methods of research on pollinators and natural enemies (Shackelford *et al*., 2013) motivated us to sample non-crop habitat as we did, within 2 km of cropland. Therefore, it is possible that ‘damage costs’ from crop raiders and livestock predators and ‘opportunity costs’ from the forgone expansion of cropland (Naidoo *et al*., 2006) could be offset by benefits from the conservation of natural habitats, such as pollination, pest control, water catchment and erosion control (Power, 2010). Indeed, the harnessing of ecosystem services for the ‘ecological’ intensification of agriculture (Bommarco *et al*., 2013) could be vital to conflict resolution, as could payments for ecosystem services, such as carbon storage (Turner *et al*., 2012; Venter *et al*., 2013).

In future research, it could also be assumed that agricultural landscapes at different distances from protected areas should have different levels of priority. For example, Noss & Harris (1986) assumed that the intensity of land use in ‘multiple-use modules’ would increase at increasing distances from core protected areas. It is not known whether there is some distance at which unprotected areas would have the strongest effects on conservation in protected areas, but some studies have assumed that areas within 25 km of protected areas would need to be ‘buffer zones’ (Wiersma *et al*., 2004; DeFries *et al*., 2005; Beaumont & Duursma, 2012). Therefore, we searched a subset of points that were < 25 km from protected areas (H3), and this caused a lot of hotspots in South America, Southeast Asia and sub-Saharan Africa to be subtracted from the strict consensus. Thus, the definition of ‘buffer zones’ could be vital to the identification of hotspots.

Similarities and differences between protected areas and the habitats that buffer them could also be vital. For example, grassland protected areas might be well buffered by a matrix of grassland habitats, but not by a matrix of woodland habitats, if these habitats differ in their habitability and permeability to grassland species (Ricketts, 2001; Wright *et al*., 2012; Cunningham *et al*., 2013) or in their ability to maintain energy flows or disturbance regimes, such as grassland fires that are started by lightning (Hansen & DeFries, 2007). The analysis based on grassland (H4) caused all of the hotspots in South America and Southeast Asia to be subtracted from the strict consensus, and the analysis based on woodland (H5) caused many of the hotspots in sub-Saharan Africa to be subtracted. Therefore, even though the hottest hotspots were found only in sub-Saharan Africa (where evidently there are significantly high proportions of both grassland and woodland surrounding cropland), parts of both South America and Southeast Asia would probably be hotspots in future research on woodland protected areas (Fig. S3).

All of the hottest hotspots, and a lot of the coldest coldspots, were in sub-Saharan Africa. This should be seen as a warning that the ‘sustainable’ intensification of sub-Saharan Africa (Pretty *et al*., 2011) should proceed only with extreme caution, because sub-Saharan Africa is a huge and heterogeneous region, in which the different subregions could have vastly different potentials for conservation conflict. If need be, the coldest coldspots could be considered hotspots for sustainable intensification, but conservation conflict should not be the only consideration, because the ‘sustainability’ of ‘sustainable’ intensification is controversial (Loos *et al*., 2014), especially in ecologically fragile subregions, such as the Sahel (Tappan & McGahuey, 2007). Central Asia, which also had a lot of the coldest coldspots, also has a history of unsustainable intensification (Cai *et al*., 2003). We need much more research on soil and water conservation (Foley *et al*., 2011; Mueller *et al*., 2012), and the regulation of agrochemicals (Jepson *et al*., 2014), before we can be confident in the ‘sustainable’ label on agricultural intensification in these regions. We also need much more research on ‘ecological’ intensification in these regions (Steward *et al*., 2014). Furthermore, we need more research at local and regional scales before we conclude that these coldspots, identified on the global scale, can safely be intensified. For example, parts of Eastern Europe that were identified as coldspots of Type I conflict in this global analysis are thought to be strongholds of agricultural biodiversity within Europe, precisely because they have not yet been intensified (Donald *et al*., 2002; Hartel *et al*., 2010), and it is possible that these parts of Eastern Europe would be identified as hotspots of conservation conflict if compared to other parts of Europe. It is possible that they might not be identified as hotspots in a global analysis because of the latitudinal gradient in species richness or because traditional methods of wildlife-friendly farming in Eastern Europe mean that many species are not yet threatened with extinction, but they could be threatened if wildlife-friendly farming is replaced by intensive farming. This points to the need for local and regional analyses and the need for proactive assumptions (assumptions about the value of biodiversity that is not yet threatened) to be incorporated into future analyses.

A recent analysis by Phalan *et al*. (2014) considered the conservation consequences of closing (or failing to close) yield gaps. This is the only other analysis (that we know of) that has considered global spatial priorities for nature conservation in agricultural landscapes. Their analysis was framed as a spatial prioritization of either intensification (closing yield gaps and thereby sparing land) or expansion (failing to close yield gaps and thus expanding cropland), whereas our analysis was framed as a spatial prioritization of either production (whether by intensifying or expanding cropland) or conservation (whether by sharing or sparing). They analysed the interactions between birds and future land use (proportion of cropland), whereas we analysed the interactions between vertebrates and present land use (proportion of non-cropland that could be cleared or degraded). There was some consensus between our analyses, and this gives us some confidence in our results. For example, in their analysis, Eastern Europe seemed to be among the highest priorities for intensification and the lowest priorities for bird conservation, and in our analysis, some of the coldest coldspots of conservation conflict were also in Eastern Europe. In their analysis, parts of the Great Rift Valley, along the African Great Lakes, seemed to be some of the highest priorities for both intensification and bird conservation, and some of the hottest hotspots of conservation conflict were also in these areas in our analysis.

When we searched for hotspots based on the top 95% of points (H2), we found a lot more hotspots than we did based on the top 98% of points (H1), and all of these hotspots could also be prioritized for conflict resolution, if time and money were unlimited. There is nothing special about a threshold of 98%, but some threshold must be used to set priorities. In this analysis, the hottest hotspots were all in sub-Saharan Africa, and it could be that our limited time and money should be spent on conflict resolution in sub-Saharan Africa, but this proof of concept should be seen as a call for research, not a call to arms for either conservation or production in either hotspots or coldspots. Moreover, there was high potential for at least one type of conservation conflict in most regions (Type I–IV heatmaps), and the global scale is only one of many scales. Furthermore, the many limitations of the underlying data sets (see Appendix S1) should be seen as a further call for research and a reason to be circumspect when drawing conclusions from our results.

In conclusion, we suggest that hotspots of conservation conflict could and should be identified as part of an ‘assessment phase’ in conflict resolution (Henle *et al*., 2008). But should we fight for nature in these hotspots, or should we cede the field to agriculture, and fight for nature where the costs are lower? To answer these questions scientifically and systematically, we could use cost–benefit analysis to optimize land use. Ethically, however, the answer is not that easy. The value of nature cannot be defined only in terms of the number of species in a landscape, and as we optimize the conservation planning system, we would do well to respect the fact that some things cannot be optimized (Fischer *et al*., 2014).

## Biosketch

**Gorm E. Shackelford** is interested in agroecology as a means of producing enough food to ‘feed the planet’ without ‘destroying the environment’. His current research is focused on habitat loss and climate change as threats to the conservation of crop pollinators and natural enemies of crop pests (especially bees and predatory wasps) in agricultural landscapes. He is also a photographer (http://www.gormshackelford.com).

## SUPPORTING INFORMATION

**Data S1** Coordinates, habitat, species, and yield for each of the 60405 cropland data points.

**Appendix S1** Supporting methods.

**Figure S1** Graphical abstract of methods.

**Figure S2** Flow chart of methods.

**Figure S3** Hotspots and coldspots from each analysis, H1–H6 and C1–C6.
