# Supplementary material for: Conservation planning in agricultural landscapes: hotspots of conflict between agriculture and nature
Source: Divers Distrib. 2014 Dec 26;21(3):357–67. doi: 10.1111/ddi.12291 (PMC4579854; doi:10.1111/ddi.12291)
Supplement: Supplementary file 2 — Appendix S1 Supporting methods. [file ddi0021-0357-sd2.pdf]

SUPPORTING METHODS

We used R (v3.0.0) (R Core Team, 2013) and QGIS (v2.0) (QGIS Team, 2014) to handle the geospatial data. In particular, we used the R packages *ggplot2*, *gstat*, *raster*, *rgdal*, and *rgeos* (Pebesma, 2004; Wickham, 2009; Bivand & Rundel, 2013; Bivand *et al.*, 2013; Hijmans, 2014) to generate random sampling points in the cropland of the world, to extract data from these sampling points, and to map the results. We used the GlobCover 2009 global land-cover map (ESA & UCL, 2010) to calculate the proportion non-crop habitat surrounding each cropland point. This map was too large to load into R and so we split it into smaller tiles (1° longitude x 1° latitude) for sampling and extracting data (Fig. S1). Within the cropland of each tile, we generated a number of random points in proportion to the area of cropland (*i.e.* we sampled with equal effort per unit area of cropland), and we then calculated the proportion of non-crop habitat within 1–4 km of each point, based on standard methods in landscape ecology (Shackelford *et al.*, 2013). We specified a minimum distance of 8 km between points, so that 4 km radii did not overlap and points were independent samples of land cover from 1–4 km. We did not see clear distinctions between the global distribution of non-crop habitat within 1, 2, and 4 km of cropland, and therefore we used the data on non-crop habitat within 2 km for all analyses.

We defined "cropland" as GlobCover classes 11 and 14 (irrigated and rainfed cropland) and a percentage of classes 20 and 30 (mosaic cropland and mosaic vegetation; see below), we defined "non-crop habitat" as classes 40–180 (grassland, shrubland, forest, *etc.*) and a proportion of classes 20 and 30 (mosaic cropland and mosaic vegetation; see below), and we did not include classes 190–230 (artificial, bare, permanent snow and ice, water, or no data classes) in calculations of land cover. The proportion of non-crop habitat surrounding a point was calculated by dividing the total area of non-crop habitat by the total area of land, not including classes 190–230 (*e.g.*, not including water), so that the results were not biased against croplands surrounded by these classes (*e.g.*, croplands on the coast). GlobCover classes 20 and 30 have variable percentages of crop and non-crop habitat. Class 20 ("mosaic cropland") is 50–70% cropland and class 30 ("mosaic vegetation") is 20–50% cropland. We defined "mosaic cropland" as 60% cropland (and thus 40% non-crop habitat), and we defined "mosaic vegetation" as 35% cropland (and thus 65% non-crop habitat), for the purposes of calculating the proportion of non-crop habitat surrounding each point. We split all non-crop habitat into either "grassland" or "woodland". We defined "woodland" as 100% of classes described as "forest" or "shrubland" (classes 40–100, 130, 160, and 170), plus 60% of class

110 (a “mosaic” class, which is 50–70% “forest or shrubland”), plus 40% of class 120 (another “mosaic” class, which is 50–70% “grassland”), plus 50% of class 180 (“grassland or woody vegetation on regularly flooded or waterlogged soils”), plus 50% of the non-crop habitat in the aforementioned “mosaic” classes (20% of class 20 and 32.5% of class 30; see above), and we defined “grassland” as 100% of classes described as “grassland or savannah or lichens/mosses” or “sparse vegetation” (100% of classes 140 and 150), plus the remainder of the non-crop habitat in the mosaic classes (classes 20, 30, 110, 120, 150, and 180; see above), such that total “non-crop habitat” = “grassland” + “woodland”.

We refer to “protected areas” throughout the text, and we mean “protected areas where restricted agricultural use is permitted” and “strictly protected areas where agricultural use is not permitted” in terms of the GAEZ definitions of these areas. These definitions were based on the World Database of Protected Areas Annual Release 2009 and the NATURA 2000 network—80% of these areas are “strictly protected” areas (*e.g.*, IUCN II National Parks), and 20% are “protected” areas with restrictions on agriculture (*e.g.*, IUCN V Managed Resource). Please see the GAEZ documentation for details (Fischer *et al.*, 2012). Clearly, there are conservation conflicts on the “agricultural frontiers” of the world, at the edge of the wilderness, such as the Amazon and Congo basins, and much of this wilderness is not protected. However, we assumed that conservation planning in agricultural landscapes would not be a replacement for protected areas. We trust that wilderness areas will be designated as protected areas when and where it is possible to do so, and they could then be included in future searches for hotspots of conservation conflict.

We used Bernoulli models in SaTScan™ v9.2 (Kulldorff, 1997, 2013) to search for hotspots and coldspots in the data points. We used SaTScan™ for several reasons. It enabled us to use unprojected coordinates (latitude and longitude), whereas many of the other methods of cluster analysis that we considered did not, and the use of projected coordinates would have resulted in unnecessary distortions to this global analysis. SaTScan™ also accounted for the density of cropland in a search area, by testing for the proportion of cases in each search area, rather than the number of cases, and this resulted in a test statistic for each search area, from which its P-value was calculated. We used the default settings in SaTScan™, except that we limited our searches to maximum areas of 100, 200, or 400 km around each point, and we set no restrictions on cluster centers (such that hotspots could overlap, and thus the maximum search areas did not restrict the size of the hotspots, because many small hotspots that overlapped could form hotspots that were larger than the maximum

search area). We used the coordinates of the data points as the centers of the search areas (the "coordinates file").

We assumed that points could be prioritized in terms of their relative values (*e.g.*, points with *c*-values that were higher than 98% of other *c*-values were cases). In future research, a balance should be found between points that would seem to be the highest priorities, because they have "superlative" values (*e.g.*, they have the highest proportions of natural habitat), and areas that would not seem to be the highest priorities, but probably should be, because they surpass an agriculturally, biologically, or ecologically meaningful threshold (*e.g.*, they have enough natural habitat to support a minimum viable population of a threatened species), even though they do not have "superlative" values.

In the GlobCover 2009 land-cover map, only about 70% of the land cover was accurately classified (Bontemps *et al.*, 2011). Nonetheless, GlobCover 2009 was the most recent and highest resolution global land-cover map that we knew of (it has a resolution of about 300 m at the equator), and therefore we suggest that it was the most appropriate map for measuring land cover within relatively small distances of cropland points (1–4 km). However, it was not possible to use this map to differentiate between plantation forests and natural forests, for example, or to differentiate between intensive grasslands and extensive grasslands or natural grasslands, and thus it is not possible to argue that the "non-crop habitats" in this analysis are "natural" or "semi-natural" habitats. Nonetheless, "non-crop habitats" are sources of heterogeneity in farming landscapes, and heterogeneity is a driver of biodiversity and ecosystem services (Benton *et al.*, 2003; Shackelford *et al.*, 2013).

The number of threatened species has limitations as a proxy for biodiversity value or vulnerability to agriculture. Only a small proportion of all species are on the IUCN Red List of Threatened Species™, only half of these species have geospatial data, and thus there could have been spatial bias in this search for hotspots, based on spatial bias in the research on threatened species. We had no data on the value of these species in terms of cultural benefits (*e.g.*, as charismatic or endemic species) or in terms of agricultural costs (*e.g.*, as crop raiders or livestock predators), and we had no data on the vulnerability of these species to agricultural intensification (data which does not exist on a global scale, except for extrapolated data on birds) (Phalan *et al.*, 2014).

The data on yield gaps are rough estimates on a coarse scale (Fischer *et al.*, 2012), and closing these yield gaps might not be possible, if investments in rural infrastructure and

agricultural inputs are not forthcoming, in which case the agricultural landscapes with the widest yield gaps might not be at maximum risk of agricultural intensification. However, these landscapes might then be at maximum risk of agricultural expansion, if the local food supply is unable to meet the local food demand. Thus, landscapes with wide yield gaps might nevertheless be hotspots of conflict between agriculture and nature.

Closing yield gaps in areas of food insecurity, or areas with high rural populations and low rural incomes, might be vital for reducing pressures on natural habitats, and data on human populations in the buffer zones of protected areas might be an important predictor of the effectiveness of protected area (Wiersma *et al.*, 2004). We did not use any sociological or economic data sets in searching for hotspots of conservation conflict. However, in Africa, where we found all of the hottest hotspots of conservation conflict, human populations are high where species richness is high (Balmford *et al.*, 2001).

Because of all these limitations, we stress that the present search for hotspots is only a proof of concept, and further research based on this conceptual framework would benefit not only from better biological data but also from economic, political, and sociological data. Furthermore, “monetized data” on biological, sociological, and economic costs and benefits should be used to complement the “non-monetized data” (“threat” and “distance-function” data) that we used in this search for hotspots (Naidoo *et al.*, 2006). Data on the cost of land in different areas would be especially useful, since the expansion of cropland could be a stronger driver of habitat loss in places with lower land costs.

SUPPORTING REFERENCES

Balmford A., Moore J.L., Brooks T., Burgess N., Hansen L.A., Williams P., & Rahbek C. (2001) Conservation conflicts across Africa. *Science*, **291**, 2616–2619.

Benton T.G., Vickery J.A., & Wilson J.D. (2003) Farmland biodiversity: is habitat heterogeneity the key? *Trends in Ecology & Evolution*, **18**, 182–188.

Bivand R., Keitt T., & Rowlingson B. (2013) *rgdal: Bindings for the Geospatial Data Abstraction Library.*

Bivand R. & Rundel C. (2013) *rgeos: Interface to Geometry Engine — Open Source (GEOS).*

Bontemps S., Defourny P., Van Bogaert E., Arino O., Kalogirou V., & Ramos Perez J. (2011) *GlobCover 2009 Products Description and Validation Report.* UCLouvain & ESA Team.

ESA & UCL (2010) *GlobCover 2009 v2.3.* European Space Agency and Université Catholique de Louvain, http://due.esrin.esa.int/globcover/.

Fischer G., Nachtergaele F.O., Prieler S., Teixeira E., Tóth G., van Velthuizen H., Verelst L., & Wiberg D. (2012) *Global Agro-Ecological Zones (GAEZ v3.0): Model Documentation.* IIASA and FAO, Laxenburg, Austria and Rome, Italy.

Hijmans R.J. (2014) *raster: Geographic data analysis and modeling.*

Kulldorff M. (1997) A spatial scan statistic. *Communications in Statistics: Theory and Methods*, **26**, 1481–1496.

Kulldorff M. (2013) *SaTScan v9.2 64-bit: Software for the Spatial and Space-Time Scan Statistics.* Harvard Medical School and Information Management Services, Inc., Boston, MA and Calverton, MD.

Naidoo R., Balmford A., Ferraro P.J., Polasky S., Ricketts T.H., & Rouget M. (2006) Integrating economic costs into conservation planning. *Trends in Ecology & Evolution*, **21**, 681–687.

Pebesma E.J. (2004) Multivariable geostatistics in S: the gstat package. *Computers & Geosciences*, **30**, 683–691.

Phalan B., Green R., & Balmford A. (2014) Closing yield gaps: perils and possibilities for biodiversity conservation. *Philosophical Transactions of the Royal Society B: Biological Sciences*, **369**, 20120285.

QGIS Team (2014) *QGIS v2.0.* Open Source Geospatial Foundation Project, http://www.qgis.org/.

R Core Team (2013) *R: A Language and Environment for Statistical Computing.* R Foundation for Statistical Computing, Vienna, Austria.

Shackelford G., Steward P.R., Benton T.G., Kunin W.E., Potts S.G., Biesmeijer J.C., & Sait S.M. (2013) Comparison of pollinators and natural enemies: a meta-analysis of landscape and local effects on abundance and richness in crops. *Biological Reviews*, **88**, 1002–1021.

Wickham H. (2009) *ggplot2: elegant graphics for data analysis.* Springer, New York.

Wiersma Y.F., Nudds T.D., & Rivard D.H. (2004) Models to distinguish effects of landscape patterns and human population pressures associated with species loss in Canadian national parks. *Landscape Ecology*, **19**, 773–786.
