# Supplementary material for: Conservation planning in agricultural landscapes: hotspots of conflict between agriculture and nature
Source: Divers Distrib. 2014 Dec 26;21(3):357–67. doi: 10.1111/ddi.12291 (PMC4579854; doi:10.1111/ddi.12291)
Supplement: Supplementary file 3 — Figure S1 Graphical abstract of methods. [file ddi0021-0357-sd3.pdf]

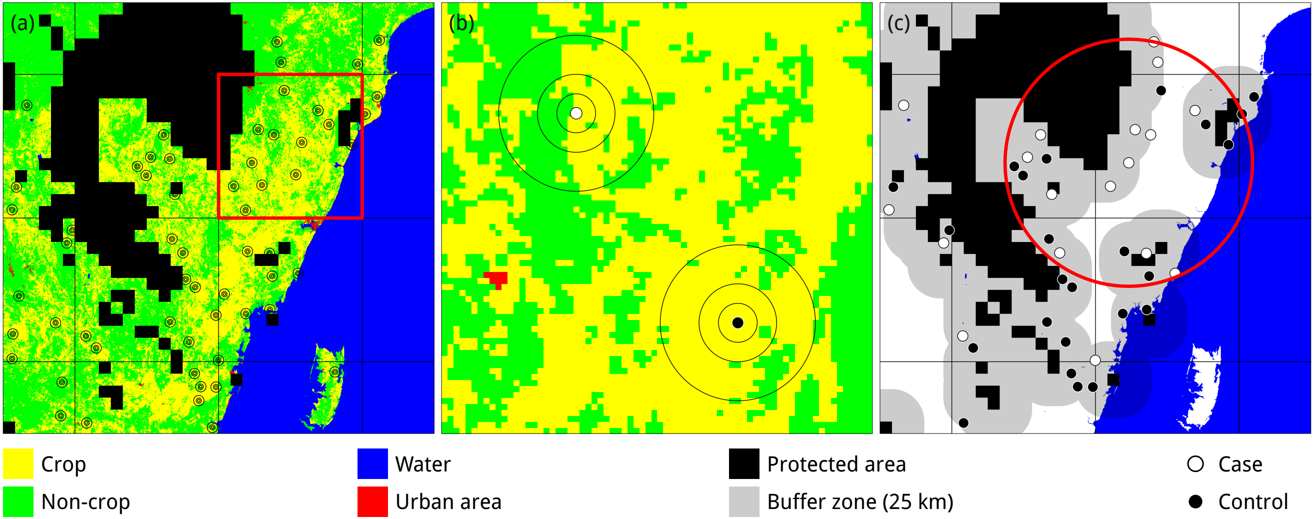

Figure S1 Graphical abstract of methods. (a) The globe was split into 1 x 1 degree tiles (*e.g.*, red square). In each tile, cropland was sampled with a number of points proportional to its area (yellow area). Points inside protected areas with restrictions on agriculture (black areas) were deleted. (b) The proportion of non-crop habitat (green area) was calculated in buffers around each point (concentric circles). The relative yield of the cropland was calculated at each point, and so was the number of "threatened" and "Near-Threatened" species of amphibians, birds, mammals, and reptiles with ranges that included that point (species with potential to live in or move through the agricultural matrix). Data from all tiles were then combined (Data S1). (c) Data points were classified as either "cases" (white points) or "controls" (black points), based on the type of conservation conflict (Table 1), and spatial scan statistics were used to identify "hotspots" in the data (areas with significantly high proportions of cases; *e.g.*, red circle). Buffer zones (gray buffers of 25 km) are shown around protected areas with restrictions on agriculture (black areas). Only the subset of data points that were inside these buffers were used in one analysis (H3 hotspots), and only the subset of data points that were outside these buffers were used in another analysis (C3 coldspots; Table 2).
