# Supplementary figures and images for: Conservation planning in agricultural landscapes: hotspots of conflict between agriculture and nature
Source: Divers Distrib. 2014 Dec 26;21(3):357–67. doi: 10.1111/ddi.12291 (PMC4579854; doi:10.1111/ddi.12291)

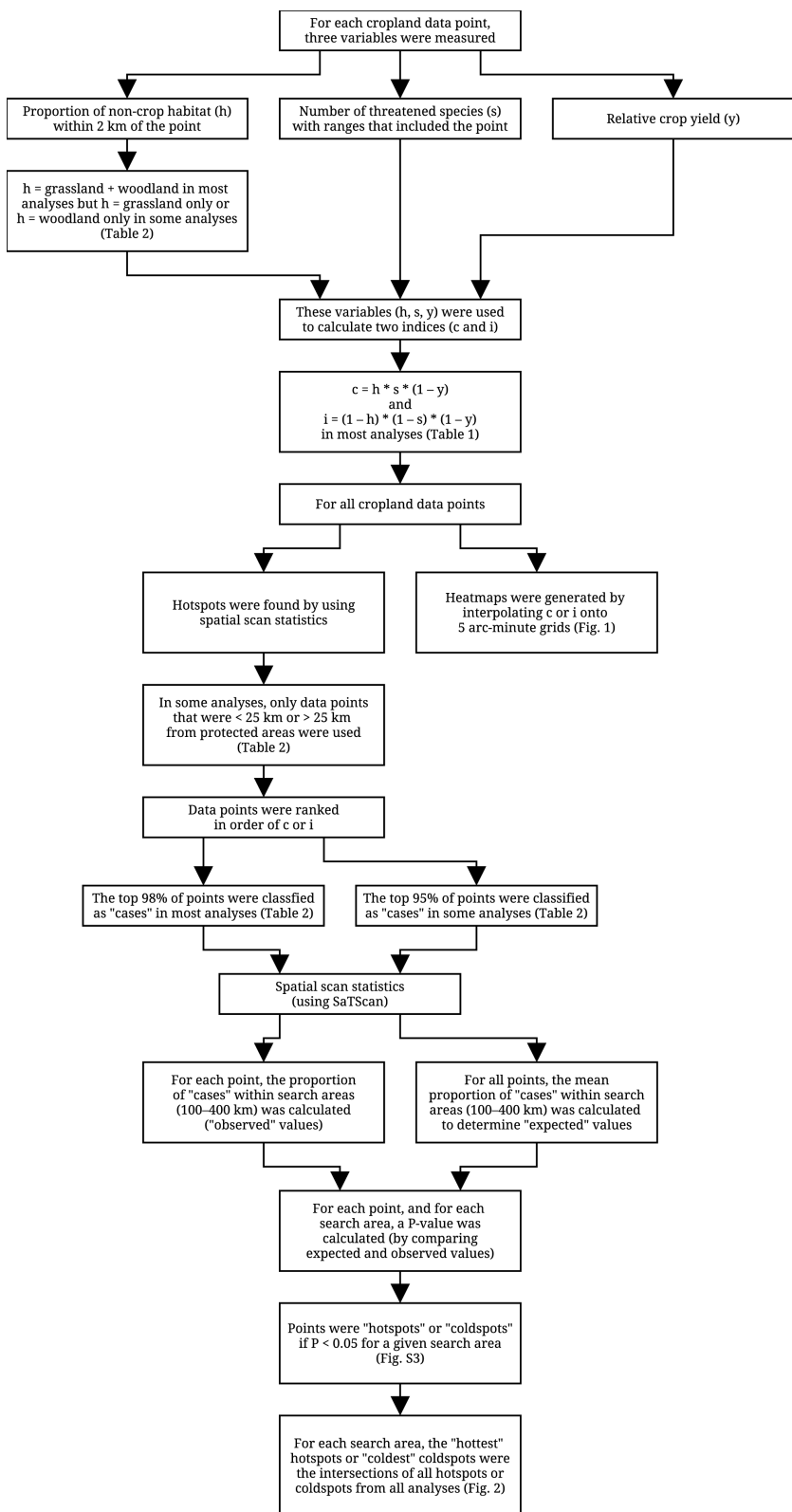

Supplement: Supplementary file 4 — Figure S2 Flow chart of methods. [file ddi0021-0357-sd4.pdf]

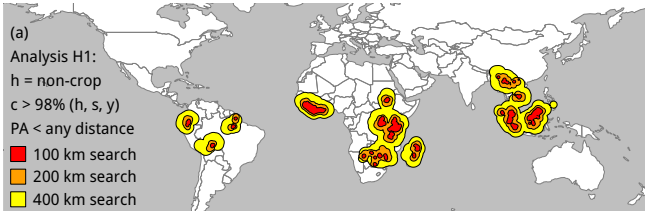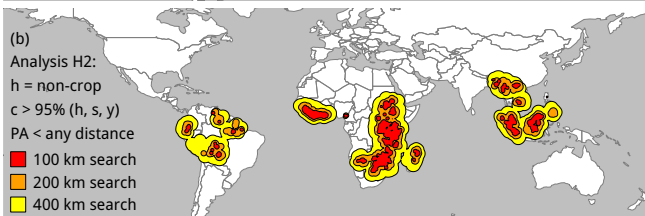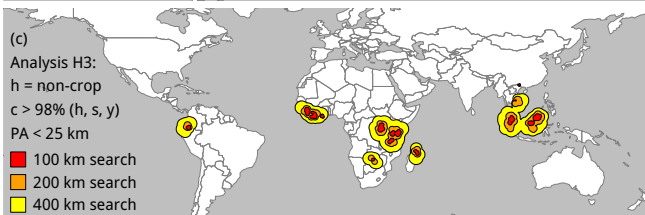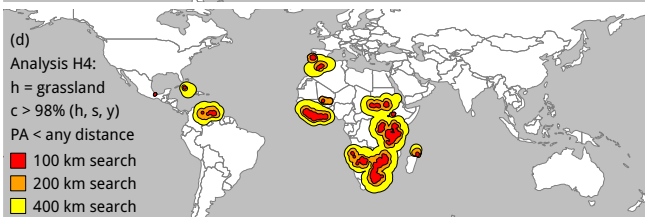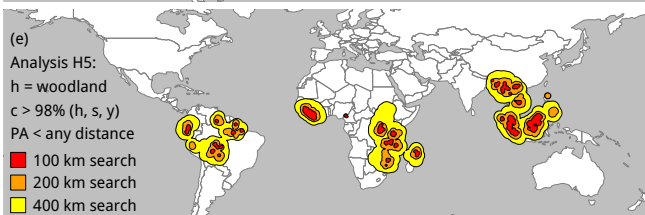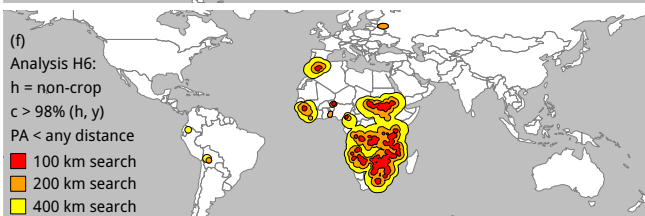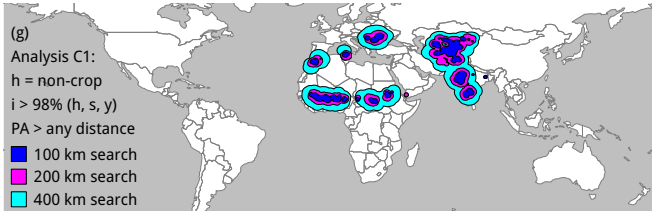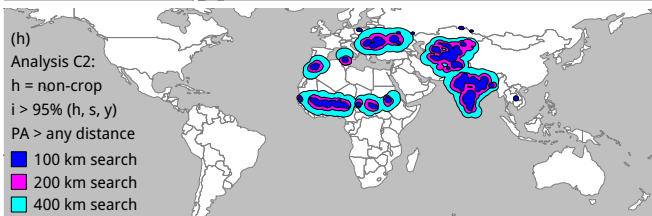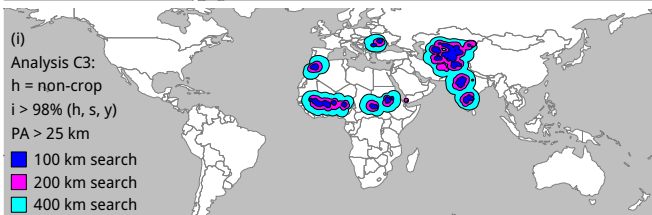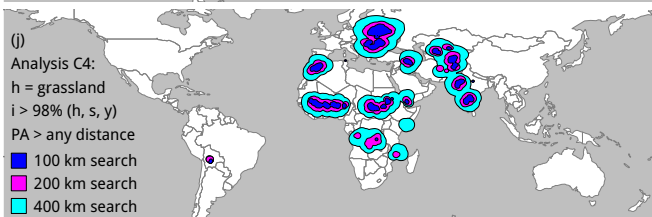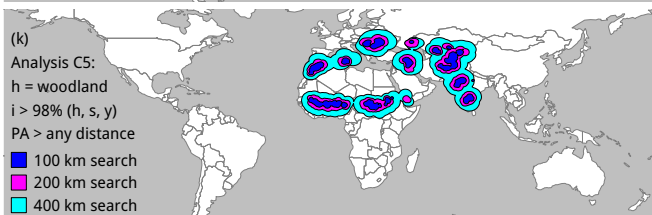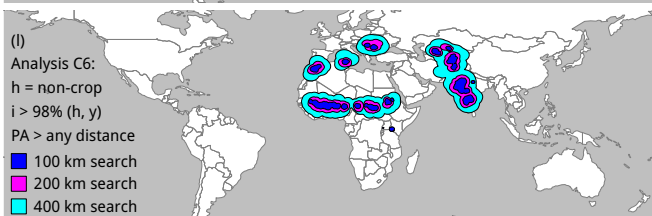

Supplement: Supplementary file 5 — Figure S3 Hotspots and coldspots from each analysis, H1–H6 and C1–C6. [file ddi0021-0357-sd5.pdf]
